# Depression and its Associated Factors among Patients with Chronic Obstructive Pulmonary Disease in Karachi, Pakistan

**DOI:** 10.7759/cureus.2930

**Published:** 2018-07-05

**Authors:** Gulshan Himani, Abida Badini, Kashmira Nanji

**Affiliations:** 1 Family Medicine, The Aga Khan University, Karachi, PAK; 2 Epidemiology and Public Health, The Aga Khan University, Karachi, PAK

**Keywords:** copd, depression, risk factors, hads, pakistan

## Abstract

Introduction

Depression in patients with chronic obstructive pulmonary disease (COPD) can be an incapacitating health problem that negatively affects the quality of life. If the depression is not treated, it is associated with increased morbidity and mortality. The goal of this study was to determine the frequency of depression in patients with COPD and examine the factors contributing to depression in these patients.

Methods

This cross-sectional study was conducted using a sample of 556 patients with COPD visiting the Pulmonology Clinic of the Aga Khan University Hospital in Karachi, Pakistan, from March 2010 to March 2011. A pretested structured questionnaire was used for data collection. The Hospital Anxiety and Depression Scale (HADS) was used to screen for depression. SPSS Statistics for Windows, Version 19.0. (IBM Corp, Armonk, NY) was used to enter and analyze data.

Results

Out of the total 556 participants, majority 62.9% were between 60 and 79 years of age and 70.1% of the participants were males. The frequency of depression in COPD patients was 57.2%, and multiple logistic regression analyses indicated being over 59 years (Adjusted odds ratio (ORadj), 2.750; 95% confidence interval [CI]: 1.25 to 6.05, p = 0.039), being male (ORadj, 2.28; 95% CI: 0.89 to 5.14), being retired or unemployed (ORadj, 1.041; 95% CI: 0.41 to 2.62, p = 0.000), using inhaled steroids (ORadj, 3.929; 95% CI: 2.59 to 5.97, p = 0.000), and living alone were significantly associated with depression in COPD patients.

Conclusion

Several risk factors for depression in patients with COPD were identified. Patients with COPD who are elderly, male, retired or unemployed, use inhaled steroids, and patients who live alone have characteristics significantly associated with depression. These factors should be considered by practicing family physicians, pulmonologists, and healthcare workers.

## Introduction

Depression affects people all over the world [1]. Patients with chronic diseases in general and those with chronic obstructive pulmonary disease (COPD) in particular are likely to have coexisting depression compared with healthy individuals. There is a higher incidence of depression in patients with more severe COPD. In Pakistan, the frequency of depression in COPD patients has been reported as 15% and 72% in two different studies, while in China, a neighboring country, the frequency of depression was reported to be 35.7% [2-4].

Depression is also associated with frequent hospital readmission for acute exacerbation and is an independent prognostic factor for mortality in such patients [5,6]. Functional impairments associated with COPD are themselves potential promoters of depressive morbidity and chronicity [7,8]. Previous studies identified many factors associated with depression in COPD patients such as age, gender, employment status, living alone, and use of steroids [9-11].

Diagnosis of depression in patients with COPD is important, as early intervention to manage depression may improve patient's quality of life and decrease the cost of management. This study aimed to estimate the frequency and risk factors of depression among patients with COPD.

## Materials and methods

This cross-sectional study was conducted on 556 COPD patients aged 40 or above, who were consecutively recruited from the Pulmonology Clinic of the Aga Khan University in Karachi, Pakistan, from March 2010 to March 2011. Patients with preexisting depression, those with a recent death (within six months) of a spouse, sibling, parent or a child, and those on oral steroids were excluded from the study.

After obtaining written informed consent, a structured pretested questionnaire was administered for data collection. The questionnaire was composed of two sections. The first section included the demographic information of the participants, and the second part included the Hospital Anxiety and Depression Scale (HADS) to screen for depression. HADS is a validated screening tool with a sensitivity of 70% and a specificity of 90% [12]. The score ranges from zero to 21 for each subscale. Those COPD patients with scores eight or higher were labeled as having depression [12].

Descriptive, univariate, and multivariate logistic regression analyses were performed using SPSS Statistics for Windows, Version 19.0. (IBM Corp, Armonk, NY). Univariate analysis was carried out using simple logistic regression to evaluate each variable for its unadjusted association with depression by computing unadjusted odds ratios (ORs) and their 95% confidence intervals (CI). Multivariable analysis was done using multiple logistic regressions to identify factors associated with depression and was reported as adjusted odds ratio (ORadj) and their 95% confidence intervals. p < 0.05 was considered statistically significant throughout the analysis.

## Results

A total of 556 of 650 patients consented to participate in the study resulting in a response rate of 85%. The demographic details of the study population are presented in Table 1. Fifty-seven percent of the patients with COPD who participated in the study were depressed.

**Table 1:** Baseline characteristics of the study participants (n = 556).

Variables	n	Percentages
Age		
40-59 Years	151	27.2
60-79 Years	350	62.9
>80 Years	55	9.9
Gender		
Male	390	70.1
Female	166	29.9
Educational status		
Not educated	41	7.4
Primary	69	12.4
Secondary	109	19.6
Intermediate	148	26.6
Higher	189	34.0
Employment status		
Employed	149	26.8
Retired	264	47.5
Housekeeper	143	25.7
Living alone		
Yes	28	5
No	528	95
Marital status		
Married	550	98.9
Unmarried	6	1.1
Household income		
<10,000 PKR	86	15.5
10,000-50,000 PKR	302	54.3
>50,000 PKR	168	30.2
Body mass index (kg/m^2^)		
<23	247	44.4
>23	309	55.6
Steroid use >3 weeks		
Yes	374	67.3
No	182	32.7
Smoking status		
Daily	115	20.7
Occasional	33	5.9
Ex-smoker	237	42.6
Never	171	30.8
Current smoking		
Never smoked	415	74.6
Smoking < 10 cigarettes/day	67	12.1
Smoking >10 cigarettes/day	74	13.3
Depression		
Yes	318	57.2
No	238	42.8

Table 2 depicts the factors associated with depression among COPD patients. After adjusting for other variables in the final logistic regression model, patients older than 59 years (ORadj, 2.750; 95% CI: 1.25 to 6.05), male (ORadj, 2.28; 95% CI: 0.89 to 5.14), retired or unemployed (ORadj, 1.041; 95% CI: 0.41 to 2.62), using inhaled steroids (ORadj, 3.929; 95% CI: 2.59 to 5.97), and currently smoking (ORadj, 3.029; 95% CI: 1.55 to 5.90) were factors significantly associated with depression in patients with COPD. People living alone had an increased risk (ORadj, 2.830; 95% CI: 0.92 to 8.73) of having depression.

**Table 2:** Factors associated with depression among COPD patients (n = 556). COPD: Chronic obstructive pulmonary disease; CI: Confidence interval; OR: Odds ratio; NS: Not significant; p > 0.05.

Variables	Depression				Unadjusted OR (95% CI)	Adjusted OR (95% CI)	p value
	Yes		No				
	n	%	n	%			
Age							
40-59 Years	64	20.1	87	36.6	1	1	0.039*
60-79 Years	208	65.4	142	59.7	6.948 (3.17-15.21)	2.329 (0.95-5.71)	
>80 Years	46	14.5	9	3.8	3.489 (1.65-7.35)	2.750 (1.25-6.05)	
Gender							
Male	238	74.8	152	63.9	1	1	0.091
Female	80	25.2	86	36.1	1.683 (1.16-2.42)	2.13 (0.88-5.14)	
Educational status							
Not educated	18	5.7	23	9.7	1	NS	
Primary	40	12.6	29	12.4	1.942 (0.98-3.84)		
Secondary	69	21.7	40	16.8	1.102 (0.63-1.92)		
Intermediate	77	24.2	71	29.8	0.881 (0.54-1.43)		
Higher	114	35.8	75	31.5	1.402 (0.90-2.16)		
Employment status							
Employed	55	17.3	94	39.5	1	1	0.000*
Retired	197	61.9	67	28.2	1.465 (0.91-2.33)	0.225 (0.12-0.40)	
Housekeeper	66	20.8	77	32.4	0.292 (0.19-0.44)	1.041 (0.41-2.61)	
Living alone							
Yes	24	7.5	4	1.7	1	1	0.070
No	294	92.5	234	98.3	4.776 (1.63-13.95)	2.830 (0.91-8.73)	
Marital status							
Married	313	98.4	237	99.6	1	NS	
Unmarried	5	1.6	1	0.4	3.786 (0.43-32.62)		
Household income							
<10,000 PKR	46	14.5	40	16.8	1	NS	
10,000-50,000 PKR	172	54.1	130	54.6	1.279 (0.75-2.15)		
>50,000 PKR	100	31.4	68	28.6	1.111 (0.75-1.63)		
Body mass index (kg/m^2^)							
<23	163	51.3	84	35.3	1	NS	
>23	155	48.7	154	64.7	1.928 (1.36-2.72)		
Steroid use >3 weeks							
Yes	260	81.8	114	47.9	1	1	0.000*
No	58	18.2	124	52.1	4.876 (3.32-7.14)	3.929 (2.58-5.96)	
Current smoking							
Never smoked	231	72.6	184	77.3	1	1	0.005*
Smoking <10 cigarettes/day	52	16.4	22	9.2	1.88 (1.32-2.02)	3.029 (1.55-5.90)	
Smoking >10 cigarettes/day	35	11	32	13.4	2.161 (1.08-4.31)	2.070 (0.91-4.67)	

## Discussion

In this study, 57.2% of patients with COPD also had depression. Two studies conducted in Pakistan reported the frequency of depression in patients with COPD to be 15% and 72%. The reason for this difference could be due to the small sample sizes used in those studies as compared to ours. Those studies also used different screening tools and were unable to control for confounding factors [2,3].

A case-control study conducted on Chinese patients with and without COPD reported that the prevalence of depression is 35.7% in patients with COPD compared to 7.2% in patients without COPD [4]. In India, 33.3% of patients with COPD showed moderate to severe depression, whereas 20.6% of patients had major depressive disorder [13]. The prevalence of depression in patients with COPD varies depending upon the country, study settings, and screening tools used.

Some studies state clinically significant levels of depression in patients with COPD were more prevalent in patients under age 60 [4,8,14]. This contrasts with our findings; we noted that patients with COPD over age 59 were more likely to have depression compared to patients aged 59 and younger. Depression in elderly COPD patients may reduce independence, resulting in a growing dependence on medical care. Similar findings have also been reported [15].

Smokers with COPD were more likely to have depressive symptoms in our study, which is a similar finding in many other studies [8,16,17].

Several studies reported gender is an insignificant determinant of depression in patients with COPD, others concluded the female gender has a significant association with depression in these patients [4,8,14,16]. In contrast, our results showed male patients were more likely to suffer from depression. This may be due to a different study population in Pakistan where men are usually the family’s main provider, and a chronic illness like COPD might cause a reduced ability to work, creating increased stress levels which can lead to depression. Further studies are needed to explore the association of the male gender and depression among patients with COPD.

We found that unemployment was directly associated with depression. Patients with COPD aged over 59 years were more likely to suffer from depression, and most of the patients over 59 years in our study were retired. A Turkish study also found similar results [9]. Living with the financial difficulties puts the retired COPD patients at a great social disadvantage and causes mental stresses and worries which then contributes toward the development of depression.

In our study, patients with COPD who lived alone were found to be at a higher risk of depression than those who have strong family support. These findings are consistent with a similar study conducted by Gudmundsson et al. on COPD and depression [10]. A possible explanation for this association of living alone and depression could be that patients living alone have fewer caregivers to provide physical and emotional support; this lack of support can contribute to depression. Interventions should be included to identify and strengthen the social networks of patients with COPD.

COPD patients on long-term steroid use reported a higher level of depression than those who were not receiving steroids. Gift et al. also found a strong association between depression in patients with COPD and steroid use [11]. Further studies are warranted to explore this relationship so that patients with COPD who also require steroid treatment can be monitored and treated accordingly.

Our study was limited in that participants of our study may not be representative of the general population; participants were subjects who attended a tertiary care hospital and may have different characteristics and severity levels than the general population. Moreover, this was a cross-sectional study, so temporality is difficult to establish, i.e., cause and effect relation between the factors and the disease (COPD).

Moreover, further studies are needed to determine the prevalence of depression in patients with COPD and to further investigate its causative factors in our society.

## Conclusions

Our findings indicate a need to focus on depressive symptoms and risk factors associated with depression among patients with COPD. Families and communities should be made aware of depressive symptoms and its associated risk factors so that patients can be screened earlier. Clinicians can support positive mental health outcomes through the early identification of patients with COPD who may be at risk for psychological distress. Clinicians could refer these patients for individual or group counseling in an effort to improve outcomes and quality of life for these patients.
